# Neuroinflammatory Pathways in the ALS-FTD Continuum: A Focus on Genetic Variants

**DOI:** 10.3390/genes14081658

**Published:** 2023-08-21

**Authors:** Fabiola De Marchi, Giacomo Tondo, Lucia Corrado, Federico Menegon, Davide Aprile, Matteo Anselmi, Sandra D’Alfonso, Cristoforo Comi, Letizia Mazzini

**Affiliations:** 1 ALS Center, Neurology Unit, Department of Translational Medicine, University of Piemonte Orientale, 28100 Novara, Italy; letizia.mazzini@uniupo.it; 2 Neurology Unit, Department of Translational Medicine, S. Andrea Hospital, University of Piemonte Orientale, 13100 Vercelli, Italy; giacomo.tondo85@gmail.com (G.T.); aprile.davide@hotmail.com (D.A.); cristoforo.comi@med.uniupo.it (C.C.); 3 Department of Health Sciences, University of Eastern Piedmont, 28100 Novara, Italy; lucia.corrado@med.uniupo.it (L.C.); sandra.dalfonso@med.uniupo.it (S.D.); 4 Neurology Unit, Department of Translational Medicine, University of Piemonte Orientale, 28100 Novara, Italy; 20042535@studenti.uniupo.it (F.M.); 20029549@studenti.uniupo.it (M.A.); 5 Interdisciplinary Research Center of Autoimmune Diseases (IRCAD), University of Piemonte Orientale, 28100 Novara, Italy

**Keywords:** genetic, amyotrophic lateral sclerosis, frontotemporal dementia, immune system, neuroinflammation, autoimmune, therapy

## Abstract

Amyotrophic Lateral Sclerosis (ALS) and Frontotemporal dementia (FDT) are progressive neurodegenerative disorders that, in several cases, overlap in clinical presentation, and genetic and pathological disease mechanisms. About 10–15% of ALS cases and up to 40% of FTD are familial, usually with dominant traits. ALS and FTD, in several cases, share common gene mutations, such as in *C9ORF72*, *TARDBP*, *SQSTM-1*, *FUS*, *VCP*, *CHCHD10*, and *TBK-1*. Also, several mechanisms are involved in ALS and FTD pathogenesis, such as protein misfolding, oxidative stress, and impaired axonal transport. In addition, neuroinflammation and neuroinflammatory cells, such as astrocytes, oligodendrocytes, microglia, and lymphocytes and, overall, the cellular microenvironment, have been proposed as pivotal players in the pathogenesis the ALS-FTD spectrum disorders. This review overviews the current evidence regarding neuroinflammatory markers in the ALS/FTD continuum, focusing on the neuroinflammatory pathways involved in the genetic cases, moving from post-mortem reports to in vivo biofluid and neuroimaging data. We further discuss the potential link between genetic and autoimmune disorders and potential therapeutic implications.

## 1. Introduction

Amyotrophic Lateral Sclerosis (ALS) is a progressive neurodegenerative disease (NDD), characterized by the degeneration of upper and lower motor neurons in the brain and the spinal cord [1]. ALS begins insidiously with focal weakness but spreads involving most muscles, including the diaphragm, usually leading to death through respiratory failure in 3–5 years [2]. ALS typically begins in the limbs (spinal phenotype), but about one-third of cases start in the cranic district (bulbar phenotype), with difficulty chewing, speaking, or swallowing. In Europe and the United States, there are 2.6 or 3 new cases of ALS per year per 100,000 people. These statistics are globally reasonably uniform, although there are rare foci in which ALS is more common [3]. About 10–15% of ALS cases are familial, usually with dominant traits, while the remaining 85–90% are sporadic. Currently, no effective treatment can revert the disease course considering that disease mechanisms and factors related to a selective vulnerability of somatic motor neurons (MNs) in ALS are not entirely understood. The last two decades of research have pushed the identification and monitoring of fluid and imaging biomarkers, aiming at revealing pathogenic mechanisms and potential therapeutic targets [4]. Since ALS and other NDDs share the deposition of misfolded proteins as a common pathogenic mechanism, a promising field of research is represented by the investigation of protein complexes and their interactions, but large-scale confirmation is still needed [5].

Frontotemporal dementia (FTD) is an umbrella definition comprising a group of NDDs characterized by progressive deficits in behavior, executive function, or language and the degeneration of the frontal and temporal lobes [6,7]. The most common phenotype is the behavioral variant FTD (bvFTD), characterized by behavioral disturbances and executive deficits. Symptoms may also include disinhibition, impulsivity, gambling, and socially inappropriate behavior [7,8,9,10,11]. Semantic dementia (SD), characterized by prominent impaired language comprehension, and progressive non-fluent aphasia (PNFA), associated with considerable difficulties in speech, are less common forms [12]. ALS and FTD can be associated in 15% of cases [13]. The clinical phenotype primarily associated with ALS is the bvFTD, although anecdotal reports describe MNs involvement associated with SD and PNFA [14]. Similar to ALS, in most FTD cases, ubiquitinated neuronal cytoplasmic inclusions of trans-active response DNA-binding protein of 43 kDa (TDP-43) are described. In 10 to 30% of FTD cases, an autosomal dominant inheritance is reported, and this percentage increases to 40% if a history of NDD is considered [15,16]. Also, ALS and FTD can share common genetic origins. Hexanucleotide repeat expansion (GGGGCC) in the first intron of the chromosome open reading frame 72 (*C9ORF72*) gene accounts for a significant proportion of autosomal dominant ALS-FTD spectrum disorders. Repeat expansions in *C9ORF72* have been identified in roughly 40% of familial ALS patients, 20% of familial FTD patients, and 6% of sporadic ALS-FTD cases [17]. In addition to the *C9ORF72* gene, other important genes associated with both diseases have been identified. Of great clinical and scientific interest remains to understand how carriers of the same genetic mutation, with the same pathogenetic mechanisms, and consequently the same protein inclusion, can express extremely different clinical manifestations.

Several mechanisms are involved in ALS and FTD pathogenesis, such as protein misfolding, oxidative stress, impaired axonal transport, alteration in RNA, mitochondrial and cytoskeletal dysfunction, and nucleocytoplasmic trafficking, with emerging evidence of a complex interaction between genetic, environmental, and molecular pathways [18]. In addition, neuroinflammatory cells, such as astrocytes, oligodendrocytes, microglia, and lymphocytes and, overall, the cellular microenvironment, have been proposed as pivotal players in the pathogenesis the ALS-FTD spectrum disorders [19,20,21,22]. Examining the role of neuroinflammatory pathways in genetic forms of NDDs may have important implications. In vitro data might improve understanding of ALS-FTD pathogenesis, focusing on specific molecular alterations [23]. Studies in mutated animals represent easily accessible and reliable models whose findings might be tested in humans. Reports on symptomatic and pre-symptomatic patients offer the possibility to identify early diagnostic alterations and mark disease progression. This review aims to provide an overview of the current evidence regarding neuroinflammatory markers in the genetic ALS/FTD continuum, moving from post-mortem data to in vivo biofluid and neuroimaging studies.

## 2. Genetic in the ALS-FTD Continuum

During the last few years, several mutations in genes related to ALS and FTD were discovered due to improvements in genetic sequencing analysis and their ease of use [24].

Superoxide dismutase 1 (*SOD1*) is the primary gene-related only to ALS development [25]. It was discovered in 1993, with more than 200 different mutations described. Although SOD1 mutations are highly heterogeneous, symptoms are often similar to sporadic forms of ALS. In some cases, patients with *SOD1* mutations present a more extended disease duration and a pure motor phenotype, generally starting from the lower limbs [26]. Other genes implicated almost exclusively in ALS are the NIMA Related Kinase 1 (*NEK1*), vesicle-associated membrane protein-associated protein B (*VAPB*), angiogenin (*ANG*), senataxin (*SETX*), and alsin (*ALS2*) [27,28,29].

Granulin (*GRN*) and microtubule-associated protein tau (*MAPT*) are the primary genes related to FTD [30,31]. *GRN* gene mutations were first identified in families with FTD (5–20%), mainly as autosomal dominant forms associated with chromosome 17. Until now, more than 120 *GRN* mutations have been identified, excluding most missense variants of *GRN* that are risk factors for Alzheimer’s disease (AD) rather than FTD [32]. Heterozygous mutations in *GRN* cause FTD, while homozygous mutations cause a lysosomal storage disorder named neuronal ceroid lipofuscinosis, suggesting that progranulin is involved in lysosomal biogenesis and homeostasis [33].

The *MAPT* gene, identified more than 40 years ago, is located on chromosome 17q21 and encodes for tau protein, a protein expressed in neurons of several brain regions and less in glial cells [34]. Until now, more than 100 *MAPT* mutations have been identified [35]. From a pathological point of view, mutations in *MAPT* occur primarily in the microtubule-binding repeat domain, and consequently, tau has less affinity to microtubules [35,36]. Other minor gene mutations exclusively associated with FTD and not ALS were found on *CHMP2B, CCNF*, and *TIA1* [15,16].

Some gene mutations are detectable in the ALS-FTD spectrum disorder and include *C9ORF72*, TAR DNA-binding protein (*TARDBP*), Sequestosome-1 (*SQSTM-1*), Fused in sarcoma (*FUS*), Valosin containing protein (*VCP*), Coiled-coil-helix-coiled-coil-helix domain containing 10 (*CHCHD10*), Optineurin (*OPTN*) and Tank-binding kinase 1 (*TBK-1*) [37,38,39,40,41,42]. The first genetic link between ALS and FTD was the discovery of the *TARDBP* mutation [43]. Mutations involving *TARDBP* act on TDP-43 protein, a DNA/RNA binding protein physiologically involved in RNA metabolism. For *TARDBP*, more than 70 different genetic variants have been described [39,41]. Mutations in this gene can act with two possible pathogenic mechanisms: the first is a depletion of this protein, leading to a loss of function, while the second is a gain of function of the aggregates in terms of toxicity [36].

The hexanucleotide repeat expansion within *C9ORF72* on chromosome 9p21 was identified in 2011, and it is responsible for a high percentage of familial cases of ALS and FTD, especially when behavioral symptoms are present [44,45]. Discovered for the first time associated with ALS-FTD cases, it has also been associated with AD, Parkinson’s Disease, atypical parkinsonism, and Huntington’s disease. Although the exact function of C9Orf72 is yet to be defined, the pathological degeneration related to this gene is provoked by several mechanisms, such as accumulation of RNA containing GGGGCC repeat in the brain and spinal cord, direct haploinsufficiency of C9Orf72 protein, dipeptide repeat protein toxicity arising from repeat-associated non-AUG translation occurring off the expansion, and disruption of nucleocytoplasmic transport [38,46,47,48,49].

*SQSTM1* gene is located on 5q35 and encodes p62, a multifunctional protein involved in various cellular functions related to neurodegenerative processes, including apoptosis, the nuclear factor kappa-light-chain-enhancer of activated B cells (NF-κB) signaling, ubiquitin-mediated autophagy, and transcription regulation. Defective p26 accumulates in aggregates, disturbing selective autophagy pathways [50].

Among the most recently discovered genes, in 2015, *TBK1* was described as associated with ALS-FTD in roughly 1% of European cases. *TBK1* is a member of the IkB kinase family involved in autophagy, mitophagy, and innate immune signaling. Most mutations are loss of function due to deletion of the C-terminal domain leading to activation of autophagy and inflammatory pathways via interferon (IFN) type 1. Even with high variability in age at onset and disease duration, clinically, *TBK1* mutations are associated mainly with bulbar onset ALS and cognitive impairment [40,51,52,53].

*VCP* represented another rare early example of gene overlapping; to date, more than 30 mutations have been reported [54]. Many of them are located on exon five within the N-terminal CDC48 domain, which is involved in ubiquitin-binding, meaning that mutations in this region may negatively affect the ubiquitin protein degradation pathway [55]. From a clinical point of view, mutations in *VCP* are associated with several disorders, Paget’s disease and inclusion body myositis among them [54]. Patients with ALS-FTD carriers of *VCP* mutations are phenotypically similar to sporadic forms [54].

Mutations in *OPTN*, encoding optineurin, are rare and associated with both neurological and non-neurological conditions, including the ALS-FTD continuum [56]. *OPTN* was identified for the first time as an ALS causative gene in 2010, and since then, more than 20 mutations have been reported. The involvement of *OPTN* in FTD is less clear, although the case series reported a percentage of 5% of FTD patients carrying the *OPTN* mutation [57]. Optineurin is a protein involved in several cellular processes that act on several pathways. Importantly, *OPTN* influences the innate immune response by negatively regulating inflammatory pathways [58].

Lastly, mutations in the *CHCHD10* gene encode for a small percentage of ALS-FTD patients. *CHCHD10* is a mitochondrial protein that regulates mitochondrial metabolism, synthesizes respiratory chain components, and modulates cell apoptosis [59]. At least 30 variants have since been reported, and they are concentrated on exon two of the gene encoding the non-structured N-terminal [59]. In addition to ALS-FTD, disorders related to *CHCHD10* mutations are several and varied, such as myopathy, Charcot-Marie Tooth neuropathy, and cerebellar ataxia [60,61].

**Figure 1:**
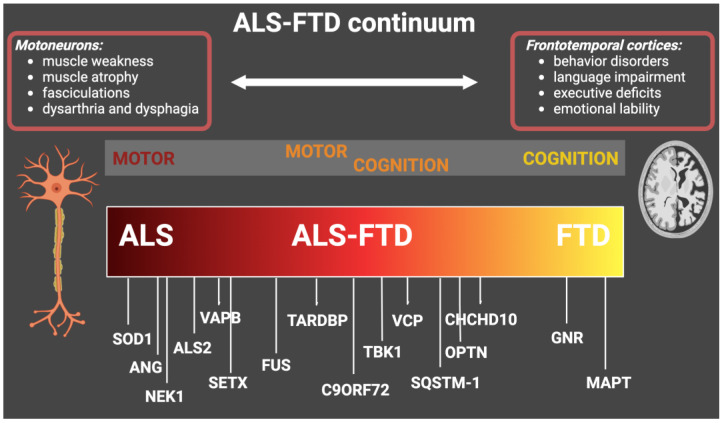
Genes with genetic mutations that cause ALS, ALS-FTD, and FTD. The figure shows the main causative genes associated with the ALS-FTD continuum and the main related clinical features. ALS: amyotrophic lateral sclerosis; FTD: frontotemporal dementia.

The involved genes along the ALS-FTD continuum are summarized in Figure 1.

## 3. Neuroinflammation in Neurodegenerative Diseases: Players and Pathways

The idea that the brain was an immune-privileged organ, primarily due to the presence of the blood-brain barrier (BBB) and the absence of a typical lymphatic system, has been overturned by the increasing evidence of the role of neuroinflammation in several neurological disorders [62]. It has been largely demonstrated the existence of a complex interplay between central and peripheral innate and adaptative inflammatory responses, which regulates brain neuroinflammation and can be triggered by various stimuli such as trauma, stroke, autoimmune disorders, infections, and NDDs [63]. CNS resident glial cells, including microglia and astrocytes, are primarily involved in protective mechanisms, aiming at repairing damaged tissue, eliminating pathogens, and removing misfolded protein aggregates and cellular debris [64]. Although initially protective, sustained activation of neuroinflammatory cells provokes detrimental consequences, resulting in neuronal dysfunction, synaptic degeneration, and neuronal death [65]. Similarly, oligodendrocytes are important players in the neuroinflammatory response, actively participating in immune-mediated processes by producing several immune regulatory factors and expressing receptors for communicating with microglia [66].

All immune cells in the CNS can contribute to the neuroinflammatory responses, both protective and deleterious. Microglia are the primary resident innate immune cells of the CNS, ubiquitously distributed in the brain, playing a crucial role in maintaining homeostasis and protecting the host against pathogenic stimuli [63]. However, several kinds of insult can activate microglia, which respond to the environment by shifting into pro-inflammatory or anti-inflammatory phenotypes and in turn sustain or switch off neuroinflammatory response, modulating the production of cytokines, interleukins (IL), and other inflammatory molecules [67]. Thus, microglia are usually classified as pro-inflammatory (M1) and anti-inflammatory (M2). M1 subtype derived from the microglia stimulation with molecules such as IFN-γ, lipopolysaccharide (LPS), or signals from damaged neurons, acting in a pro-inflammatory environment with increased levels of IL-1β, IL-6, IL-12, IL-18, and tumor necrosis factor (TNF)-α. On the contrary, M2 cells operate in an anti-inflammatory environment with the predominant release of IL-4, IL-10, IL-13, and the Transforming Growth Factor (TGF)-β. These two opposite states represent only the polar extremes of microglia activation, since microglia can react to different types of neuronal injury throughout the expression of multiple phenotypes, and are able to modify the microenvironment secreting specific factors [68,69].

Astrocytes are the most representative cells in the brain, contributing to brain homeostasis and neuronal support, by providing energy metabolites and regulating extracellular ionic balance. In addition, astrocytes modulate synaptic activity and regulate BBB permeability, favoring or limiting the infiltration of other inflammatory cells [63]. Similar to microglia, astrocytes can be activated by various types of stimuli and can provide a neuroprotective response, switching off neuroinflammation and stimulating tissue repair, or promoting inflammation and neuronal damage [70]. Two main astrocyte subpopulations have been well characterized: the pro-inflammatory phenotype (A1), producing neuroinflammatory factors such as IL-1a, TNF-α, and complement protein 1q, and the immune-modulatory phenotype (A2), which play neuroprotective functions by producing neurotrophic factors and anti-inflammatory cytokines, such as IL-4, IL-10, TGF-β [71]. However, multiple phenotypes can be expressed depending on the environmental balance. Astrogliosis’s beneficial or detrimental effect is again related to the type of stimulus, the specific disease and its phase, and the involved molecular pathway [63].

The T-lymphocytes primarily mediate adaptive immunity in the CNS. As for the other CNS inflammatory cells, the environment and the levels of several cytokines and chemokines influence the expression of anti-inflammatory or pro-inflammatory phenotypes. Type 2 helper lymphocytes (Th2) and T regulatory (Tregs) cells are involved in anti-inflammatory and regulatory activities, while Type 1 (Th1) and Type 17 (Th17) cells are essentially pro-inflammatory [72]. In addition, dysregulation of cytotoxic CD8 T cells has been revealed in patients with several NDDs [73].

## 4. Neuroinflammatory Pathways in the Genetic ALS-FTD Continuum

### 4.1. Post-Mortem and Preclinical Evidence

As in other NDDs, also in the ALS-FTD spectrum, the deposition of misfolded proteins is one of the disease hallmarks, leading to progressive MNs and neuron damage. Misfolded protein deposition is associated with the activation of neuroinflammatory cells and pathways. ALS post-mortem studies have reported that neurodegenerative changes involving upper and lower motor neurons are associated with neuroinflammatory responses and the activation of inflammatory cells, including microglia, astrocytes, oligodendrocytes, and lymphocytes. In post-mortem examination of the brain and spinal cord of ALS patients, reactive microglia have been shown in the affected areas, including the primary motor cortex and the anterior horn of the spinal cord [74]. In addition, T-lymphocyte infiltrates have been detected in the spinal cord of ALS autopsy cases, with T cytotoxic cells most abundantly expressed in the ventral horns [75]. Similarly, a widespread cortical neuronal and extensive glial TDP-43 aggregation in motor and extramotor regions was described in ALS patients, including C9ORF72 mutated patients [76,77]. Post-mortem studies on genetic ALS/FTD patients are limited, and a few works have profiled the glial role. ALS and FTD C9Orf72 patients, despite being clinically different, are pathologically similar: both exhibit p62+ granules, dipeptide repeat protein deposits, and RNA-binding protein TDP-43 aggregates in CNS. In a recent study, Rifai and colleagues profiled the morphological and spatial glia-related markers, including the ionized calcium-binding adapter molecule 1 (IbA1), expressed by macrophages and microglia, the CD68 and FUS staining in C9Orf72 post-mortem tissues. Microglia markers were accurate classifiers of disease status (motor phenotype) and correlated with pathological and in vivo clinical features of C9Orf72-ALS patients, sustaining the hypothesis of the role of microglia in determining clinicopathological features [78]. The link between neuroinflammatory responses and pathology may depend on the genetic variant. A recent post-mortem immunohistochemistry report of a case of TBK1 mutated patient established a classical ALS and type B TDP pathology without changes in TBK1 staining or interferon regulatory factor-3 (IRF3), a transcription factor that controls multiple IFN-inducing pathways related to inflammation and immunity. These data may suggest that the TBK1 loss of function mutation did not have negative effects on IRF3 expression or localization [79].

Further insight can be derived from animal models. Animal studies allow us to explore the impact of pathological alterations on the neuroinflammatory environment, the relationship between protein deposition and glial activation, and the clinical repercussions. The most important protein aggregates in ALS include the SOD1, TDP-43, and FUS [80]. TDP-43 deposition plays a crucial role in ALS neuroinflammatory responses and pathogenesis. Extracellular TDP-43 activates microglia-enhancing pro-inflammatory pathways such as the NF-κB, which is involved in MN death [81]. NF-κB is a transcriptional factor associated with the toll-like receptor (TLR) pathways, modulating the inflammatory response and favoring the expression of pro-inflammatory cytokines such as IFNγ, IL-1, IL-6, and TNF-a [82]. Also, FUS represents a critical activator of the NF-κB pathway, leading to MN apoptosis induced by TNF. In the study of the group of Pasinelli, in nontransgenic mice, the effect of the primary astrocytes expressing an N-terminally GFP tagged R521G mutant or wild-type FUS was evaluated, showing that mutFUS-expressing astrocytes undergo astrogliosis, damage co-cultured MNs via activation of an inflammatory response and produce a conditioned medium that is toxic to MNs in isolation [83]. However, most evidence regarding microglia activation in ALS pathogenesis is related to SOD1. The SOD1 mouse model is the most used in ALS preclinical research [84]. The NF-κB is upregulated in the spinal cord of SOD1 mice, and its constitutive activation in microglia provokes gliosis and motor neuron death [85]. The critical role of a dysregulated inflammatory response is underlined by the evidence that SOD1 mutations involving only neurons are insufficient to precipitate the disease in ALS mouse models, while mutations ubiquitously expressed, thus also involving glial cells, cause a rapidly progressive and fatal disease [86]. SOD1 mutations induce microglia and astrocyte activation and overexpression of the IL-1β, which has been related to ALS progression [87]. Coherently, in the SOD1 mouse model, the gene deletion or suppression in glial cells increases survival [20,88]. SOD1 mutation mice also show altered astrocyte activity, whose pro-inflammatory phenotype overproducing TGF-β has been associated with accelerated disease progression [89]. Similarly, T-cells and especially regulatory T-lymphocytes (Tregs) are considered neuroprotective in ALS, thus a T-cells deficiency has been associated with decreased survival [90,91].

As for FTD, progranulin knockout mice are particularly interesting since they show impairment in social behavior without impact on motor and memory functions [92]. This model provided evidence for increased astrogliosis and microglia activation [93]. Several transgenic TDP-43 mouse models have been described, which can develop a progressive and fatal NDD with both features of ALS and FTD with ubiquitinated aggregates [94]. Similarly, a mouse model with C9ORF72 mutation and concomitant motor deficits and features of ALS/FTD has been described [95]. Even if both depletion of TDP-43 and C9Orf72 have been associated with the clearance of pathological aggregates by potentiating microglial activity [96], this boosted clearance comes at a cost, and depletion of microglial TDP-43 also results in enhanced synaptic loss [97] while the C9Orf72 deficiency has been shown to promote microglia-mediated alteration in learning and memory behaviors in mice [98].

If animal models have the advantage of being able to highlight alterations of the inflammatory/immune system already in the preclinical disease phase, post-mortem studies, which for obvious reasons capture the terminal phase, cannot discriminate the appearance of the immune alterations, but only document their presence. In this regard, in recent years, biofluid investigations measuring inflammatory markers and imaging studies with tracers able to detect microglia activation have been developed to highlight pathological changes in vivo.

### 4.2. In Vivo and in Human Studies

#### 4.2.1. Fluid Biomarkers

Fluid biomarkers can be indexes of pathophysiological processes and can be used for diagnostic or prognostic purposes, to stage diseases, or to monitor therapeutic responses [99]. In the field of neurodegenerative dementia, one of the main aims of neurodegeneration markers dosage in the cerebrospinal fluid (CSF) is to differentiate AD from other dementia, including FTD. Low amyloid-β42 levels, low amyloid-β42/amyloid-β40 ratio, and high tau/amyloid-β42 ratio are indicative of AD pathology [100]. Conversely, FTD is likely the main cause of dementia in patients with high total-tau CSF levels and normal amyloid-β levels [101]. Plasmatic dosages of amyloid-β and tau protein are currently under evaluation and, in FTD, high total-tau levels have been reported [102]. In addition to classical markers for AD pathology, one of the more studied biomarkers in serum and CSF is the neurofilament light chain (NfL), which represents neuron-specific markers of axonal damage, despite lacking disease specificity and being detected in a wide range of NDDs. Much evidence shows that high levels of NfL are present both in CSF and in the serum of ALS and FTD patients when compared with controls [103] and that they could be interpreted as a diagnostic and prognostic biomarker [104,105]. Specifically, compared with other neurological disorders and normal controls, ALS patients showed higher NfL levels [106], directly correlating with clinical severity and disease progression [107]. Similarly, FTD patients, compared with other NDDs such as AD, showed higher CSF NfL levels [108], and NfL dosage helped to distinguish FTD from FTD phenocopies (subjects without signs of neurodegeneration, e.g., no frontotemporal atrophy in MRI, FDG-PET hypometabolism and stable serial clinical and neuropsychology assessment over time) [109]. In addition, plasma NfL levels in bvFTD patients directly correlated with imaging markers of neurodegeneration, including white matter degeneration and cortical thickness [110]. Regarding the role of NfL in genetic patients, a geno/phenotype-specificity in NfL release has been hypothesized by a recent work showing higher plasma NfL levels in GRN ALS patients compared with C9Orf72, and lower plasma NfL levels in C9Orf72 with slowly progressive disease [111]. The evidence of NfL elevation in pre-symptomatic carriers added prognostic relevance to the measurement in genetic patients [111]. Similar results have been reported in another study investigating CSF and serum NfL in pre-symptomatic carriers and patients with MAPT, GRN, and C9ORF72 mutations, revealing higher NfL levels in the symptomatic stage, correlating with neurodegenerative imaging changes, clinical impairment, and survival, and with good correlation between CSF and serum measurements [112]. Finally, an interesting work by Huang and colleagues, exploring the relationship between NfL and disease progression in ALS, supported the hypothesis of a link between neuroinflammation and neurodegeneration. The authors reported higher NfL plasma and CSF levels in ALS patients compared with controls and in the C9Orf72 subgroup compared with other ALS patients [113]; in addition, NfL levels correlated with faster disease progression and were associated with increased levels of inflammatory cytokines and chemokines, such as monocyte chemoattractant protein (MCP-1) and IL-18, confirming the prognostic role for NfL and suggesting a relationship between neuronal damage and neuroinflammatory responses [113].

Further fluid biomarkers studies focused on specific markers of neuroinflammation [114,115]. Chitotriosidase (CHIT1) and chitinase-3-like protein 1 (YKL-40) are neuroinflammatory markers and can be measured in biological fluid. CHIT1 is mainly expressed by myeloid cells, while YKL-40 is expressed by reactive astrocytes and microglia [116,117]. Although in a preliminary phase, CHIT1 and YKL-40 appear to be raised in symptomatic genetic FTD compared to asymptomatic mutation carriers and controls, suggesting that neuroinflammatory cell activities manifested over the symptomatic disease phase [118]. In addition, the expression of proteins involved in neuroinflammatory responses might be associated with the clinical phenotype. In a study evaluating genetic carriers of the C9ORF72 mutation and manifesting an ALS or an FTD phenotype, ALS patients showed higher CSF levels of CHIT1 and neurofilament medium polypeptide (NEFM), and the ubiquitin carboxyl-terminal hydrolase isozyme L1 (UCHL1, an enzyme related to neuronal development) levels were upregulated in ALS patients, while neuronal pentraxin receptor (NPTXR, a protein operating as a trans-synaptic organizer) levels were downregulated in FTD [119]. These aspects can suggest that different processes of ubiquitination and autophagy in the ALS and FTD phenotypes can be present. As a confirmation, glial fibrillary acidic protein (GFAP), a marker of astrogliosis, has been measured in several genetic mutations, including C9ORF72, GRN, and MAPT, and showed to be increased only in symptomatic GRN mutation carriers, correlating also with lower cognitive scores and brain atrophy [120]. Other works reported additional neuroinflammatory markers in GRN patients: several cytokines, chemokines, and markers of glial activation have been reported to differ in GRN patients compared to other mutation carriers, including IL-15, the RANTES (Regulated upon Activation, Normal T Cell Expressed, and Presumably Secreted) chemokine, and several TNFs [121].

Lastly, the protein triggering receptor expressed on myeloid cells 2 (TREM2) is an immune receptor expressed by microglia which is upregulated in neuroinflammatory responses to neuronal injury [122]. Increased CSF levels of the soluble form of TREM2 have been initially associated with multiple sclerosis [123]. However, a large amount of data suggests the involvement of TREM2 in AD and other neurodegenerative dementia [124]. In autosomal dominant AD, increased CSF levels of TREM2 have been associated with amyloid plaque deposition [125]. Nevertheless, TREM2 mutations have been associated with the development of pre-senile FTD-like dementia [126,127]. In a study evaluating CSF soluble TREM2 levels in patients with genetic FTD including different mutations, only patients carrying GRN mutation showed higher levels than controls, and TREM2 values correlated with total tau as a marker of neuronal injury [128].

Beyond generic markers of neuronal damage and inflammation, several specific biomarkers serve to identify patients carrying pathogenic mutations [99]. Patients carrying the GRN mutations show reduced levels of CSF and blood progranulin levels, which makes this dosage highly accurate and less expensive than genetic testing, mainly when performed in blood [129,130]. TDP-43 pathology, accumulating in neurons and glial cells in ALS/FTD, is associated with cell death, finally causing increased TDP-43 levels in CSF and blood [131]. However, its specificity may be debatable, being associated with other tauopathies [132].

#### 4.2.2. Neuroimaging Biomarkers

Neuroimaging, including brain magnetic resonance imaging (MRI) and 18F-fluorodeoxyglucose-positron emission tomography (FDG-PET), plays a crucial role in the diagnostic workup of FTD patients and brain atrophy involving frontotemporal regions and regional hypometabolism have been included in the current diagnostic criteria for bvFTD, SD and the PNFA [9,10,133]. Similarly, PET imaging of brain tau deposition was a precious tool in identifying regions involved in the pathological process in FTD and other tauopathies, correlating with neurodegenerative changes and clinical impairment [134,135]. Even though the role of neuroimaging in ALS is still debated due to non-univocal findings [136,137], several structural and molecular imaging studies proposed brain MRI and FDG-PET as reliable measures of neurodegenerative changes in both sporadic [138,139,140] and genetic patients [141,142,143].

PET imaging of neuroinflammation is a unique tool to in vivo reveal neuroinflammatory responses, which have been constantly reported in several NDDs and different phases of neurodegeneration [144,145,146]. The 18-kDa translocator protein (TSPO), an outer mitochondrial membrane protein, is the gold standard for detecting inflammation and gliosis, being overexpressed on activated microglia and astrocytes [147]. Again, studies employing PET imaging of neuroinflammation reported consistent results in FTD patients, while in ALS the data are less conclusive.

Since the early studies in FTD, the topographical distribution of microglia activation visualized using [^11^C]PK11195, the first and most widely used TSPO-PET tracer, colocalize with the distribution of neuronal damage and pathology, thus including frontal, prefrontal, and temporal cortices [148,149]. These findings have been confirmed in genetic patients in an early work involving only three individuals, pre-symptomatic gene carriers with the missense N279K MAPT gene mutation, leading to the FTD-parkinsonism linked to chromosome 17. Microglia activation was present mainly in the frontal cortex but also involved in the occipital and the posterior cingulate cortex in pre-symptomatic carriers compared with controls, suggesting neuroinflammatory responses as an early disease marker at the prodromal stage [150]. As shown by biofluid marker studies, revealing that neuroinflammatory markers may differ according to the genetic subtypes, neuroimaging studies also reported different microglia activation patterns in several mutation carrier subtypes. In the work of Lant and colleagues, the distribution and extent of microglia activation were compared between FTD and AD cases and controls. FTD cases showed higher frontal tracer uptake than AD, and interestingly, tracer binding was higher in temporal regions in patients carrying the MAPT mutation than GRN and C9ORF72, suggesting that the different neuroinflammatory patterns may be helpful in in vivo identifying specific genetic cases [151]. Subsequent investigation added clinical and pathological confirmation to the role of neuroinflammation in driving neurodegeneration. The group of Rowe and colleagues at Cambridge University (UK) provided the most notable contribution in this field with several elegant investigations. The relationship between microglia activation, clinical phenotype, and pathology was initially investigated in 31 patients with FTD and different clinical subtypes, including bvFTD, SD, and PNFA. The study showed a distinct spatial distribution of microglia activation, correlating with tau deposition (visualized by tau-PET) and TDP-43 pathology (revealed by the post-mortem exam) in the different FTD syndromes. Furthermore, the study also included six genetic patients: in the bvFTD group, the three C9Orf72 patients had higher [^11^C]PK11195 binding than sporadic, while two bvFTD patients with MAPT mutation and the PNFA patient with GRN mutation showed no significant different binding than sporadic. The three C9Orf72, two MAPT, and two GRN cases were further investigated and described in a subsequent study. Even with the limitation of a small sample, the results suggested that the distribution of neuroinflammation may reflect the clinical heterogeneity contributing to the development of the different clinical syndromes, as confirmed, for instance, by the prominent frontostriatal microglia activation in the bvFTD with MAPT mutation, while the PNFA with GRN mutation showing left inferior frontal inflammation [152]. Lastly, an association between neuroinflammation and clinical decline has been proposed. In the same cohort of FTD patients, Malpetti and colleagues reported a significant association by baseline microglia activation in the frontal lobe, detected by the [^11^C]PK11195-PET, and the estimated rate of cognitive change, suggesting that neuroinflammation may accelerate neurodegeneration, and supporting the potential for immunomodulatory treatments in NDDs [153].

**Figure 2:**
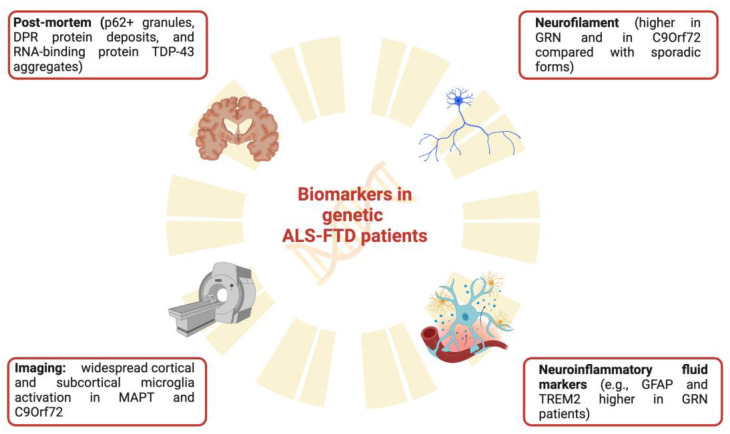
Biomarkers in genetic ALS-FTD. The figure shows the main biomarkers in the ALS-FTD continuum, divided into post-mortem, neurofilament, neuroinflammatory fluid biomarkers, and imaging. ALS: amyotrophic lateral sclerosis; FTD: frontotemporal dementia.

PET imaging of neuroinflammation has been used in ALS, but fewer data are available in genetic patients. Overall, studies investigating microglia activation in sporadic ALS and employing TSPO tracers reported widespread microglia activation in several brain regions, including the motor, prefrontal cortex, and extramotor regions including the temporal lobe, and subcortical structures [154,155,156]. In particular, microglia activation has been related to in vivo brain volume reduction and white matter alterations and, as a consequence, to clinical symptom severity [155,157,158]. In addition, a study combining MRI spectroscopy and PET imaging of neuroinflammation using one of the most used second-generation TSPO tracers, the [^11^C]-PBR28, showed in forty subjects with probable ALS that metabolic markers of gliosis and neuronal injury correlated with microglia activation in the precentral gyri [159]. These data again support the association between neuroinflammation and neurodegenerative changes, which can reflect clinical impairment. To date, only one study investigated microglia activation in genetic ALS patients. The study employed PET imaging of neuroinflammation using the [^11^C]PK11195-PET in four asymptomatic and six symptomatic mutation carriers of the SOD1 mutation. Both symptomatic and asymptomatic SOD1 mutated subjects showed significant and widespread cortical and subcortical microglia activation compared to healthy subjects. However, ALS symptomatic patients showed marked microglia activation in the motor, prefrontal cortices, and somatosensory regions, suggesting a pattern of progression of neuroinflammatory responses from the asymptomatic to the symptomatic phase [160]. These data, even needing further confirmation, support the crucial role of PET imaging of neuroinflammation in staging the disease progression and in testing novel therapeutic strategies targeting neuroinflammatory mechanisms and monitoring response to therapy. The main findings regarding clinical biomarkers involved in genetic ALS-FTD patients are summarized in Figure 2.

## 5. Autoimmune Disorders and Systemic Inflammation in Genetic ALS/FTD

Several clinical studies focused on the epidemiology of sporadic ALS and FTD support the idea that patients affected by these disorders have a higher incidence of autoimmune diseases. In 2013, the group of Turner evaluated a large UK cohort with autoimmune diseases (e.g., asthma, celiac disease, young-onset diabetes, multiple sclerosis, and systemic lupus erythematosus), reporting in this group a higher percentage of ALS compared to the general population and hypothesizing the possibility of shared genetic and/or environmental risk factors [161]. Although the data is less evident in cohorts of sporadic FTD patients [162], an interesting paper investigated the association between immune disorders and patients with mutations in the *GRN* gene, observing a significantly increased risk of autoimmune disorders clustered around inflammatory arthritis, cutaneous and gastrointestinal disorders in the PGNR cohort [163].

Regarding other selected cohorts of genetic patients, more results regard the C9Orf72 patients. Firstly, in 2016, in a US cohort, it was reported a high prevalence of autoimmune diseases (similar to what was described for GRN patients) in C9Orf72 ALS/FTD patients compared to controls and other sporadic neurodegenerative patients [164]. A recent multicentric Italian cohort study described 150 ALS C9Orf72 patients, in which an increased incidence of autoimmune diseases was not observed, whereas the authors reported a longer survival in C9Orf72 patients with ALS and thyroid disorders [17]. Looking at the problem in reverse, genotyping a small cohort of patients with ALS and multiple sclerosis, 80% of patients with both neurological conditions carried a C9Orf72 mutation. In the same report, they described an imbalance in the immune pathway, with an NF-κB activation and a concomitant CXCL10 downregulation in the CSF [165]. The presented studies and the possible correlation between NDDs, genetics, and autoimmunity are supported by the recent detection of *TBK1* mutations in the ALS/FTD spectrum. In fact, as we have mentioned, *TBK1* plays a crucial role as the hub for many innate immune signaling pathways [166].

Also, while systemic inflammation is overall present in ALS/FTD patients, it is not clear if these phenomena are a disease consequence or have a causative role. As global markers of inflammation and peripheral immune disbalance, several recent studies focused on alterations in peripheral markers, such as the neutrophil-to-lymphocyte ratio, in ALS and FTD. Alterations in this ratio, and others related, were associated with poorer prognosis and shorter survival by several groups [167,168,169], but in no study, the genetic component is considered. Also, the studies that analyzed the C-reactive protein in ALS did not primarily focus on genetic subgroups of patients [170,171].

## 6. Therapeutic Implications

Correctly understanding the pathogenetic mechanisms underlying NDDs is a cornerstone for setting up pharmacological clinical trials aimed at acting on the involved altered pathway. In this regard, the study on patients’ carriers of genetic mutations is beneficial. The target objectives of clinical trials carried out by researchers may differ. However, in the case of trials reserved for single groups of genetic patients, the common aim is reducing the production of the mutated gene (which can be, for example, mRNA or proteins). Various pharmacological therapies may be implemented for patients with a gene mutation, including antibodies related to mutated genes, agents targeting pathological mRNA sequences of mutant genes, or “more traditional” agents acting on the main pathological pathway mutation-specific. Although in a preliminary phase for most genes and with some concerns regarding short- and long-term safety, some trials with potential therapeutic agents such as single-chain antibodies and antisense oligonucleotides are ongoing.

For the primary gene involved in the ALS/FTD spectrum disorders, the *C9ORF72*, ongoing interesting clinical trials are acting on inflammatory systems. For example, Al Therapeutics started a phase II trial (NCT05163886) with a molecule called LAM-002 (apilimod dimesylate), a first-class highly selective PIKfyve kinase inhibitor. Although the results of phase IIa are not yet published, the company has announced exciting findings, namely the increased expression of the target biomarker (the glycoprotein nonmetastatic melanoma protein B) and the substantial reduction of the toxic (poly(GP)) aggregates [172]. Another interesting clinical trial involves using AL001, a recombinant human anti-human sortilin (SORT1) monoclonal IgG1. Sortilin is a type I membrane glycoprotein abundantly expressed in the CNS. As a sorting receptor in the trans-Golgi network, it is involved in neurotrophin signaling, lysosomal degradation, and APP metabolism. After a trial on FTD-GRN patients in June 2018, this antibody received orphan drug designation from the US Food and Drug Administration for the FTD treatment. In 2019, an open-label, multi-dose Phase 2 study in people with *C9ORF72* mutations causative of FTD started, but due to the COVID-19 pandemic, only a small number of patients were recruited. However, interestingly, a reduction in NfL was observed in this small group. Based on these preliminary results, in 2021, a trial for C9Orf72 ALS and FTD patients started (NCT05053035), but no results are currently released. Lastly, although without a primary autoimmune mechanism of action, also following the encouraging results of tofersen in SOD1 ALS patients, a phase 1b/2a trial (FOCUS-C9, NCT04931862) recently tested the effect of antisense oligonucleotides (WVE-004) on C9Orf72 ALS-FTD patients. However, in March 2023, the trial was discontinued because WVE-004 did not show clinical benefit compared with the placebo, and also, the reduction in (poly(GP)) unfortunately did not correlate with any clinical outcomes. Similar results were obtained for another ASO, the BIIB078 (NCT04288856), which failed to reach the outcome. Moreover, a phase III trial with ASO (ION363, NCT04768972) is ongoing for FUS ALS patients. However, in this trial, the drug selectively targets one mutant, P525L, which is responsible for an aggressive juvenile form of ALS [173]. To date, no other clinical trials have been ongoing for genetic patients that focus on the immune/inflammatory system or other damage mechanisms.

## 7. Conclusions

ALS and FTD are two severe neurodegenerative disorders of adulthood that greatly impact the patient, their family members, and society. No available therapies for both diseases are able to change the disease course. Regarding ALS, riluzole is currently the only approved drug able to delay progression by a few months, regardless of the disease stage. The discovery of several genes, including *C9ORF72, TARDBP, SQSTM-1, FUS, VCP, CHCHD10*, and *TBK-1*, associated with the ALS/FTD continuum, and the consequent detection of new disease mechanisms, including those related to inflammatory/autoimmune responses, is allowing the development of new potential therapeutic strategies applicable to the entire cohort of patients. Identifying neuroinflammation as a crucial player in the pathogenesis of NDDs has opened new diagnostic, prognostic, and therapeutic perspectives. Genetic studies identifying pathogenetic mutations in genes with immune function, such as *TBK-1,* may be crucial to understanding if neuroinflammation represents the initial trigger of neurodegeneration. Neuroinflammatory markers, especially when easily detectable in biofluids such as blood and CSF, may be specific for different genotypes and associated with corresponding phenotypes [118,128]. Similarly, neuroimaging studies of neuroinflammation may aid in identifying brain distribution patterns of microglia activation specific to a peculiar genotype [151]. The pattern of microglia activation, when detected in vivo, may help in staging the disease progression, and in familial cases, in the future, it may serve to intercept asymptomatic cases [150,160]. Thus, neuroinflammation could be crucial in both sporadic and familial disease cases, and since dysregulation of the immune system can represent a disease trigger, acting on the immune response might be a critical strategy in customized therapeutic approaches. The genetic research may add to the ALS-FTD patients’ management the possibility of identifying novel specific gene-targeting therapies, representing a decisive step into the challenge against neurodegenerative changes, potentially extending perspectives to sporadic cases.

## Data Availability

Not applicable.
